# Can we identify general architectural principles that impact the collective behaviour of both human and animal systems?

**DOI:** 10.1098/rstb.2018.0253

**Published:** 2018-07-02

**Authors:** Alan Penn, J. Scott Turner

**Affiliations:** 1 The Bartlett School of Architecture, Faculty of the Built Environment, University College of London, London, UK; 2 Department of Environmental and Forest Biology, SUNY College of Environmental Science & Forestry, Syracuse, NY, USA; 3 Stellenbosch Institute for Advanced Study, Stellenbosch, South Africa

**Keywords:** biomimetic, stigmergy, niche construction, extended cognition, space syntax

## Abstract

The search for general common principles that unify disciplines is a longstanding challenge for interdisciplinary research. Architecture has always been an interdisciplinary pursuit, combining engineering, art and culture. The rise of biomimetic architecture adds to the interdisciplinary span. We discuss the similarities and differences among human and animal societies in how architecture influences their collective behaviour. We argue that the emergence of a fully biomimetic architecture involves breaking down what we call ‘pernicious dualities’ that have permeated our discourse for decades, artificial divisions between species, between organism and environment, between genotype and phenotype, and in the case of architecture, the supposed duality between the built environment and its builders. We suggest that niche construction theory may serve as a starting point for unifying our thinking across disciplines, taxa and spatial scales.

This article is part of the theme issue ‘Interdisciplinary approaches for uncovering the impacts of architecture on collective behaviour’.

## 1. Introduction

When René Descartes formulated ‘mind–body’ dualism, his aim was to distinguish humanity from the animal world, and at the same time to salvage a place for God in a material world. For the Cartesian, the duality is between mind and body—between the transcendent mind and the clockwork machinery of living things. This distinction, and the form of logical argument upon which it was based, has been taken up enthusiastically by a large strain of modern biological thought, which has sought to impose a host of its own dualities, among them the duality between organism versus environment, between phenotype versus genotype, categorical species from categorical species [1]. This has left a history of the biological and social sciences that is strewn with paradoxes [2]. As we do with the Cartesian duality between mind and body, we might ask whether the dualities that permeate modern biological thought help or hinder our understanding. Arguably, modern biology has yet to grapple critically with this question, which has led modern biology into a philosophical crisis [3].

Duality is also rearing its head in the emerging trend of biomimetic architecture [4]. Like other forms of biomimicry, biomimetic architecture is motivated by a desire that human architecture should draw inspiration from the constructions of ‘animal architects’, such as the remarkable structures built by social insects [5,6]. The implication is that there is a fundamental duality between animal architects and the human variety. That we should draw inspiration from ‘designs’ in nature rests upon a Darwinian argument: that natural selection has shaped these structures to levels of optimality and economy that human architects would do well to emulate [7].

However laudable the inspiration, it is worth remembering that modern Darwinism is permeated with its own pernicious dualities: of genotype versus phenotype, of organism versus environment, to name two, which has left the landscape of modern Darwinism strewn with its own paradoxes [3]. These ramify into biomimetic architecture's own dualities: that human architecture is somehow radically distinct from other ‘architectures’ in nature, and that there is a fundamental distinction between the built environment and the agents that build it. Here, builders (agents) construct an environment in which the builders live. The built environment becomes thereby a ‘machine for living’, an object external to its inhabitants, that works to enable the inhabitants to live comfortably. The building does so by controlling flows of matter and energy between the environment and inhabitants so that the inhabitants' physiological needs can be met: suitable flows of heat, fresh air, moisture and information. As a Cartesian might argue that the body is a machine that houses a separate and transcendant soul, so the biomimetic architect casts the building as a machine that houses a separate and living being.

Add to this the materialistic logic that pervades modern Darwinism, and the perniciousness only deepens: a machine (a building) is designed to mimic another machine (an inhabitant). This is the sterile logic of Le Corbusier and the Bauhaus: machines to house interchangeable machines [8]. Which brings us directly to the question: what is biomimetic architecture intended to mimic? Is it living ‘machines’, or is it the unique phenomenon of life itself? This, in turn, prompts another question: if there *is* something distinctive about living nature that transcends the machine metaphor of life, what precisely would that be? And what are the prospects for incorporating it into architectural practice? Is there something beyond the building-as-machine metaphor?

## 2. Cognition, the extended mind and the extended organism

Arguably, what makes living systems unique is that they are *cognitive* systems [3,9,10]. Machines, in contrast, cannot be cognitive systems. While machines can imitate some aspects of cognitive agency (the *raison d'être* of artificial intelligence), they cannot be cognitive systems in the same way living systems are. For example, some aspects of cognition, such as constructing sensory representations, processing the information in those representations and acting upon them, are certainly amenable to automation. Yet cognition also includes phenomena such as intentionality and creativity [11], which seem less amenable to reduction to computation. Living cognition in full seems tied inextricably to the uniquely living phenomenon of mind. In short, living cognitive systems are a kind of embodied mind.

In the cognitive sciences, embodied cognition was introduced as a theoretical complement to traditional dualist interpretations of mind and body [12]. Broadly stated, the theory holds that cognition is shaped by an organism's body in such a way that sensory and perceptual systems, as well as the motor systems, determine how concepts and categories are formed and influence reasoning and problem-solving (e.g. [13–16]). Closely related to this is the notion of extended mind and situated cognition. These address how organisms manipulate the environment in service of cognition to offload and scaffold cognitive processes [14,15,17–19]. Stated most broadly then, embodied cognition and extended mind are important theoretical contributions to our understanding of how brain, body and environment interact, in that they explain how sensorimotor capabilities, embedded in some natural context, determine thought and action. For a review of developments across this line of theorizing, the reader is referred to Newen *et al.* [20] and their compendium of papers on embodied, embedded, enactive and extended cognition.

These notions of ‘embodiment’ and the ‘extended mind’ now form the dominant paradigm in cognitive science. The same logic can be extended to swarms of autonomous agents, so-called swarm cognition, which forms the basis of many theories of emergent systems, such as organisms and organism-like systems (for example, social insect colonies) or ecosystems [21–23]. In contrast to most evolutionary thought, which draws a sharp distinction between organism and environment, theories of embodied mind treat organism and environment as interactive and inseparable. Not only do organisms work and evolve to fit into an environment (adaptation), organisms actively modify the environment to suit themselves: adapting the environment to the organism. This dissolves one of biomimetic architecture's pernicious dualities: environment and organism are inseparable, two aspects of a single phenomenon: adaptation.

Turner has argued that adaptive systems are necessarily cognitive systems [3,11]. An adaptive system must *know* the state of the relationship between organism and environment, and *know* what work must be done to bring both into coherency. This is goal-directed intentional behaviour (the literal meaning of the term ‘adaptation’: toward aptitude), and it undermines biomimicry's self-imposed duality between human architecture and ‘natural’ (i.e. non-human) architecture. If adaptive systems are necessarily cognitive systems, and if adaptation is the driver of evolution, the tip of the evolutionary spear, so to speak, then there is no distinction to be drawn between human builders and animal builders. The constructions of both are reflections of a universal cognition that distinguishes all life.

For Turner, this goal-directedness is embodied in a radical interpretation of Claude Bernard's concept of homeostasis [23]. What follows logically from this is Turner's notion of the extended organism, which abolishes the duality between organism and environment: the environment is as ‘alive’ as the organism inhabiting it [1]. This opens up a new metaphor for the notion of biomimetic architecture [24]. Now, rather than speaking of the building-as-machine, we may speak of the building-as-organism, a vital extension of the organism's own physiology. Now, the built environment and organism are partners in a physiological conspiracy (they literally ‘breathe together’), both dynamically adaptable and ever-shifting, all serving the same fundamental aim of homeostasis [25]. The physiological conspiracy drives the adapted form forward in time, its persistence serving as a kind of evolutionary fitness.

Humans, along with social insects (bees, ants, some wasps and termites) provide some of the most dramatic examples of this physiological conspiracy, and it has obvious relevance for biomimetic architecture: what if our constructions mimicked the dynamic interaction of organism, environment and built environment that occurs routinely in nature?

In this paper, we investigate the parallels that can be drawn between social insects and humans in their adaptation of the environment through building, and the way that in turn these artificial environments support the social interactions of their communities or colonies. We ask whether there is an underlying theoretical framework through which the building behaviour of these different phyla and their social-functional interactions can be unified.

We develop our discussion in three stages. First, we look at the logical restrictions on what it is possible to construct. Next, we look at the effect of different sensory and bodily competencies on shaping organisms' interactions between each other and their environment. We consider what this might imply for differing cognitive models of their environment—the ‘umwelt’—of different species, and the different possible social structures that might be perpetuated thereby, through the constraints imposed by configuring the environment and its interaction with individual and group behaviours. Finally, we discuss the role of the built environment in the reproduction and evolution of social forms across phyla. This last, we propose, may help account for human social and cultural evolution proceeding at a rate far faster than genetic evolution, while also accounting for social forms outlasting, on occasion, the lifespan of the individuals from which they are composed.

## 3. The logic of the constructible

The act of construction can be thought of as inherently simple while, at the same time, giving rise to the largest and most complex of human artefacts. Piling stones upon one another, for example, is a primitive repetitive building technique. Out of this repetition can emerge buildings of exquisite design, as in the mortarless walls of the Great Zimbabwe [26]. By what logic can the repetitive act of piling stones upon one another connect to the sublime structures of the Great Zimbabwe? We argue that it is impossible to do without an appreciation of the cultural and cognitive agency of its builders [27,28].

Are humans unique in their ability to create a Great Zimbabwe out of the repetitive process of piling stones upon one another? Arguably, no. In the world of social insects, for example, constructed nests can also be extremely large and spatially complex in their construction, rivalling human constructions in complexity and beauty. Social insect nests, too, may be constructed by the repetition of many relatively simple repeated actions. Individual insects may pick up grains of sand, transport them somewhere, and lay them down again, either glued in place with a kind of mortar, or simply laid down with a dollop of saliva. We ask: how does this simple and repetitive act produce the sublime architecture of the leaf-cutter ant nest [29,30]? As with the Great Zimbabwe, there seems to be an inseparable cognitive and cultural dimension to the construction of the social insect nest, even as the context is radically different.

What logical inference should be drawn from this? For example, should we highlight the dissimilarities of context—form and function—and conclude that humans and leaf-cutter ants are radically different forms of architect? Or should we highlight the similarities—the similar processes of construction—and conclude that leaf-cutter ants and humans are the same kinds of architect? In short, what is the logic of the constructible environment? We explore this question through three examples: Guy Theraulaz's agent-based model, exemplified by the constructed nests of the termites *Apicotermes*; Scott Turner's notion of the extended organism and swarm cognition, developed in a different termite species, *Macrotermes*; and Bill Hillier's conception of beady-ring settlements in the South of France.

## 4. Agent-based model

Lijie Guo, Guy Theraulaz and colleagues describe a simulation of termite nest building, focusing on the remarkable nest of the termite genus *Apicotermes*. The *Apicotermes* nest is made up of a spiralling series of galleries cut through by ramp-type structures and columns. Guo, Theraulaz and colleagues show that the nest's characteristic helicoidal and linear ramps can result from a particular suite of cellular building processes [31].

**Figure 1.**
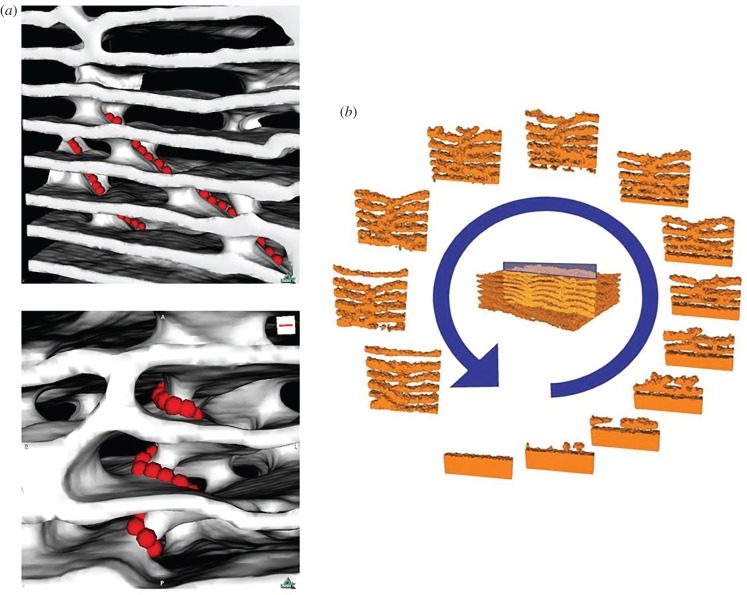
The construction of the helicoidal ramp structure of the *Apicotermes* nest. (*a*) (top) Ramps (visualized by red dots) and (bottom) a helix. (*b*) Simulation of the nest construction dynamics (figures 2 and 5 from Guo *et al.* [31]). Reprinted with permission from Guo *et al.* [31] (Copyright © 2016 IEEE). (Online version in colour.)

Their simulation entailed termites whose building behaviour consists of moving earth from the floor to the ceiling of the galleries. They show that changing the parameters for a model for nest construction by *Lasius niger* ants [32] can create the characteristic helicoidal structure of the *Apicotermes* nest. The constructing agents, the individual termites, are therefore agents whose behaviour is governed by simple rules of interaction, with other agents programmed with the same rules, and with the built environment they create (figure 1).

The *Apicotermes* nest is a marvellous example of how multitudinous agents driven by simple behavioural rules of repetitive aggregation can produce objects of great beauty and complexity. Yet is it appropriate even to speak in such terms? Is there an aesthetic of nest construction that governs the behaviour of the *Apicotermes* swarm? In the Darwinian metaphor for biomimetic architecture, the answer to this question must be ‘no’: it is functional effectiveness—adaptation of some form—that is the driver. *Apicotermes* nests therefore exist in their present form because nest form has been refined by generations of natural selection from ancestral species with more primitive nests [33,34]. The evolutionary trajectory toward the *Apicotermes* nest's present sublime form has been driven by incremental accrual of fitness advantage.

It has proven difficult to identify what these advantages might have been, however. Schmidt attributed the form to superior ventilation and protection from predators [33,34], with superior nest ventilation being the favoured explanation [35]. There is little evidence to support that claim, however. Theraulaz and colleagues have modelled the emergence of the *Apicotermes* nest using principles of self-organization and self-assembly [36,37]. He attributes the emergence of complex nests to the greater complexity of information exchange and versatility of behaviour that accrues to increases in the colony population [38,39]. In this sense, it is versatility *per se* that has been the fitness advantage.

We might reconstruct Theraulaz's ‘logic of the constructible’ in this way. Construction is explained by rules of interaction of building agents, with both other building agents and the structures that they build. Increased complexity arises from the nonlinear dynamics of the interaction between agents and the environment they build [40–42]. There remains at the heart of this logic another pernicious duality: function is divorced from structure. The aesthetic of the *Apicotermes* nest is an accidental outcome of these evolving rules of interaction, producing structures that garner selective advantage through some unidentified adaptation.

## 5. The extended organism and the aesthetic of niche construction

A different approach is Turner's extended-organism idea [1,43], which came out of his work on the mounds built by the fungus-cultivating termites of the Macrotermitinae, specifically the mound-building termites of the genus *Macrotermes*. Turner builds on Odling-Smee, Laland and Feldman’s notion of niche construction. Where a conventional interpretation of Darwinian evolution holds that organisms evolve to fit their environmental context, niche construction theory [44] recognizes that as often as not organisms actively adapt their immediate environment to suit their own needs. The extended-organism idea is the physiological dimension of Dawkins’ notion of the extended phenotype [45]. This idea treats the mound as essentially a superorganismal organ of physiology, as much alive as the termites that build it. This incorporates a directed dynamism to the mound structure, with a much more fluid interaction with the environment than strictly agent-based models, like those of Theraulaz and Bonabeau, allow [38]. So, for example, Turner and colleagues have identified several drivers of mound building and disassembly [46], which relegates the concept of stigmergy (in Grassé's original sense of the term: 1959 [36]), centrally important to the self-organization concept, to a limited context of mound repair. Such variation can be modelled using agent-based algorithms, as Jost *et al*. [47] have done for the influence of air currents on architecture of ant nests. In the extended-organism metaphor, however, variation of mound architecture arises from a rich interplay of building behaviour and the cognitive environment enclosed by the built structure. The difference is, in part, philosophical: cognition embodies a kind of striving that agent-based models do not consider [11].

**Figure 2.**
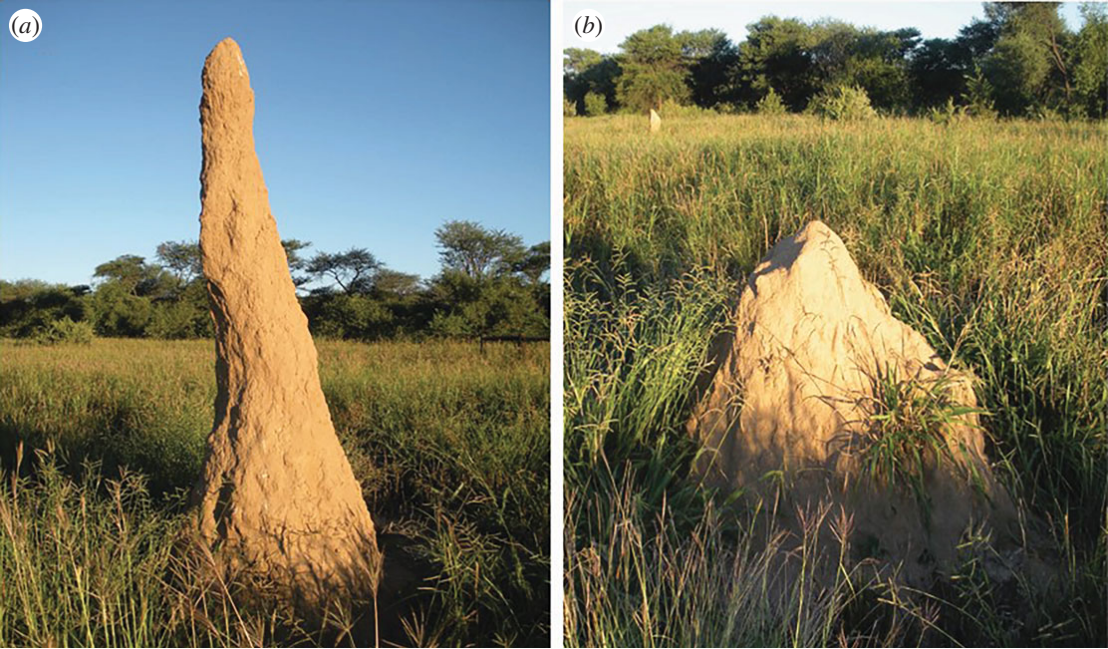
Characteristic mound architecture of two common *Macrotermes* species (*a*) *M. michaelseni* and (*b*) *M. natalensis* (Photo credit J. S. Turner). (Online version in colour.)

So, for example, two species, *Macrotermes michaelseni* and *Macrotermes natalensis*, each build distinctive mounds: *M. michaelseni* builds conical mounds topped by a tall spire while *M. natalensis* build conical mounds without the spire. These differences in mound architecture can be attributed to differences in swarm cognition: *M. michaelseni* construction is influenced more strongly by water transport and regulation of nest moisture, while *M. natalensis* construction is influenced more strongly by stigmergy and mound repair (figure 2).

So far, this conception is fully consistent with the agent-based model of nest construction (e.g. [47]). However, the cognitive dimension of the extended-organism idea embodies an aesthetic of construction. The source of the aesthetic dimension is homeostasis, the signature idea of the nineteenth century contemporary of Darwin, Claude Bernard. Bernard was a physiologist, not an evolutionist, architect or student of social insects. Yet Bernard's conception of homeostasis colours our interpretation of all these fields.

Bernard regarded homeostasis as life's fundamental property, that which distinguishes life from non-life (the 1927 reprint of Bernard's 1865 *An Introduction to the Study of Experimental Medicine* [48]). This is an essentially vitalist idea that is quite at odds with our modern conception of homeostasis. Our modern tendency is to reduce homeostasis to cybernetics, to elucidate the mechanisms that produce regulation of a specific property of the body, say, temperature. Bernard's own conception of homeostasis turns the cybernetic idea on its head: whereas the cybernetic conception regards homeostasis as the outcome of mechanism, Bernard regarded mechanism as being the outcome of homeostasis.

**Figure 3.**
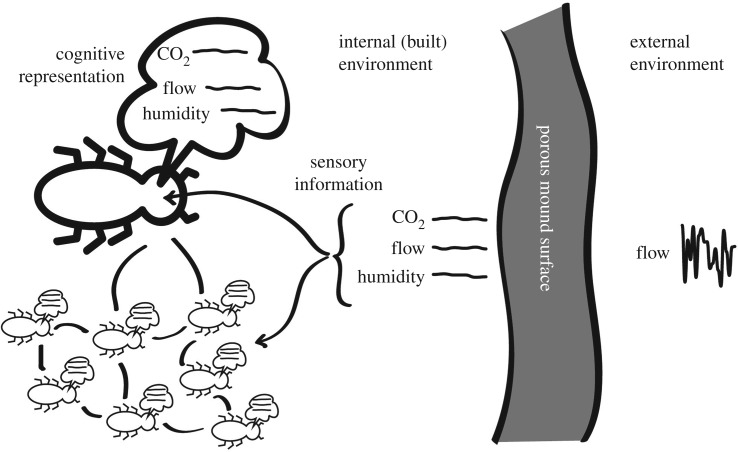
The cognitive world of the *Macrotermes* extended organism. Termites form a cognitive representation, at both individual and swarm level of a ‘desirable’ environment of steady environmental conditions. These conditions are the result of a constructed environment based upon the engineered porous interface of the mound surface, which acts to filter energy in turbulent external winds.

Among other things, Bernard's conception of homeostasis broadens the scope of the phenomenon of cognition to include the ability to shape environments according to some mental representation. This is where the distinction from automata-based models emerges. While swarms of automata can shape environments, they cannot properly be said to *want* to shape environments in a particular way: they are machines acting out an algorithm. In living cognitive systems, while mental representations of the environment often reflect the environment, they need not do so, and when they do not, cognition comes to embody intentionality and creativity, both a kind of wanting [49]. Homeostasis, in the Bernardian sense, opens the door to novelty and appeal: an aesthetic, in short (figure 3).

**Figure 4.**
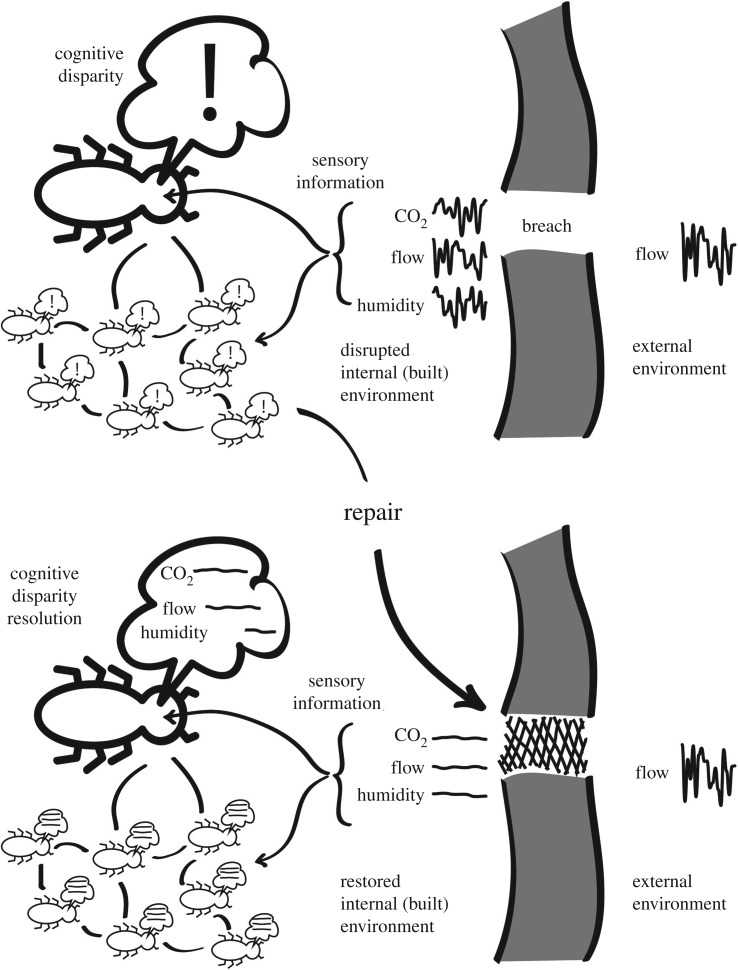
Mound-environment homeostasis as resolution of cognitive disparities. A breach in the mound disrupts the internal environment of the mound, which leads to a cognitive disparity at both individual and swarm levels. This initiates a programme of mound repair, which ultimately resolves the cognitive disparity.

In *Macrotermes,* this essentially aesthetic tendency is revealed in the phenomenon of mound repair. An environment that ‘appeals’ to termites includes still air, and steady concentrations of oxygen, carbon dioxide and water vapour (humidity). An ‘unappealing’ environment is marked by unpredictable and rapid changes in the environment: slight gusts, fluctuations of oxygen and carbon dioxide concentrations, or humidity. These disturbances usually come about in the aftermath of damage to the structured boundary of the mound, which is porous and modulates the effects of the windy and turbulent external environment. The response of termite swarms to any disparity in appeal is to mobilize a colony-wide project to reshape the environment to restore the colony environment to an appealing state. This includes ongoing colony-level decision-making, which can persist over several months, even years, far beyond the lifespan of any individual worker termite [46] (figure 4).

In this instance, the duality between organism and environment dissolves. The termites, nest and mound constitute a physiological conspiracy to co-opt and tame environments to the colony's aesthetic demands.

One can object to the notion of termites, or any agent-based self-organized system, being motivated by aesthetics. Occam's razor, for example, would seem to favour the simpler explanation of rules of interaction between essentially robotic agents and their environments (in the sense of Jost *et al.* [47]), without introducing complications such as aesthetics. It is a valid point of difference, and a frankly philosophical one, delineated by the question: can one explain the behaviour of any living system without accounting for life's fundamental attributes of cognition, homeostasis and striving [2,3]? It is worth noting that Occam's razor is not simply an appeal to favour the simplest possible hypothesis: it is an admonition to not generate complex hypotheses *without necessity* [50]. We argue that cognition, and all that is implied by that, is just such a necessity. Without accounting for that, there can be no unifying principles that explain the built environments of organisms ranging from insects to humans.

## 7. Beady-ring settlements

Bill Hillier and colleagues' approach to human settlements was different [51]. They aimed to develop an account of the vast array of human settlement forms found in the archaeological and anthropological record. They developed an ideographic language in which elementary generators such as a carrier space, the relation of containment and a boundary, are brought together in a logical syntax. They show how this language can be used to express, in a greatly simplified way, the main spatial features of a wide range of different built forms. This is the ‘syntax’ of space syntax. At its simplest level, they show how a process of rule that restricted random aggregation of buildings generates the spatial characteristics of a class of what they called ‘beady-ring’ settlements [51].

**Figure 5.**
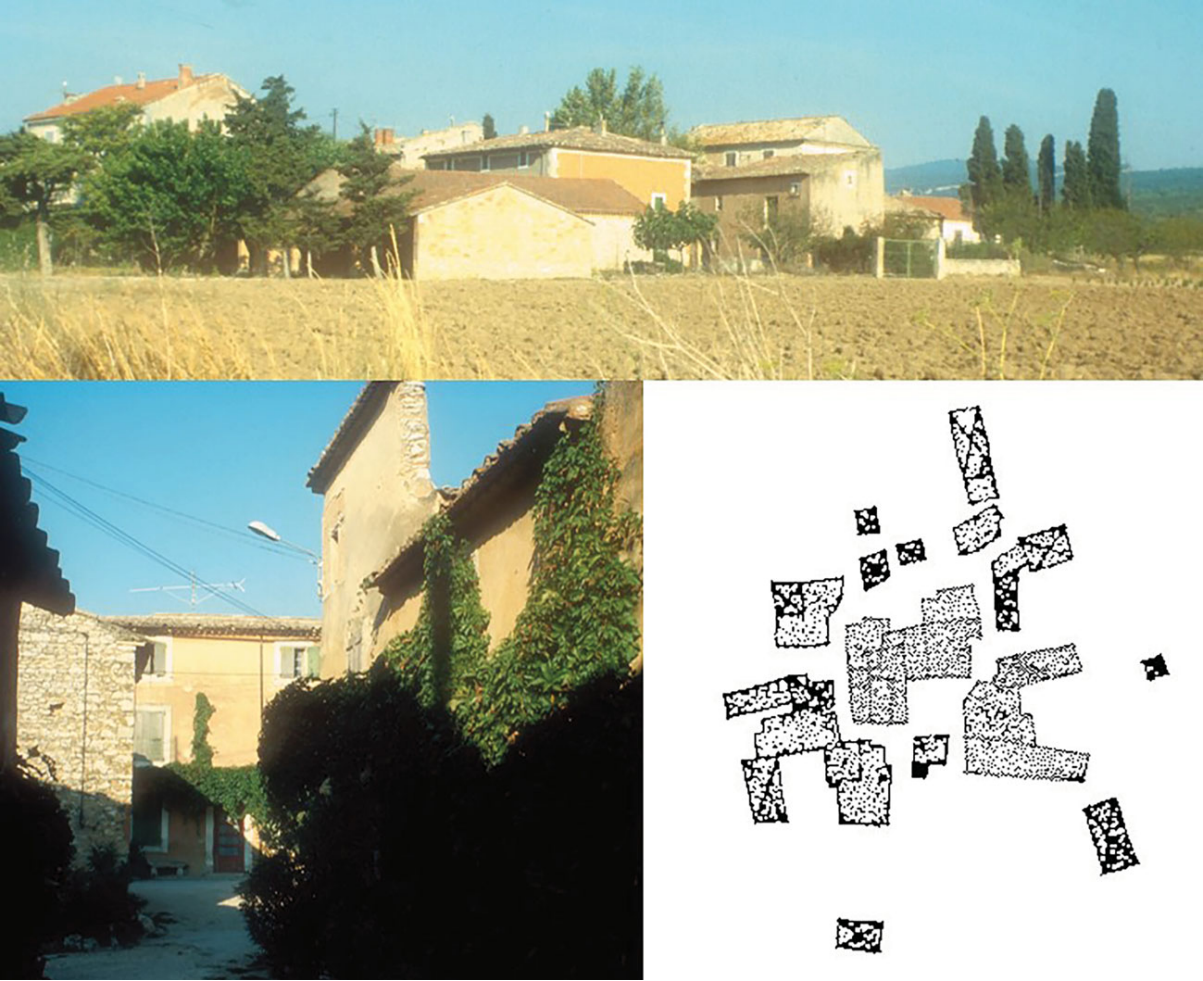
Beady-ring settlements (photo credit Bill Hillier), Map of Les Yves 1961 (figure 9 from Hillier & Hanson [51], p59). Reprinted with permission from Hillier & Hanson [51] (Copyright © 1989 Cambridge University Press). (Online version in colour.)

The beady-ring settlements Hillier describes occur in the Vaucluse region of Southern France, and are small hamlets characterized by an aggregation of houses usually forming a ring of circulation around a central clump of buildings, with several routes out to the surrounding countryside. The ring of circulation is composed of wider and narrower spaces—the ‘beads’ on the ring—and has the property that all locations are directly overseen by entrances to the houses (figure 5).

**Figure 6.**
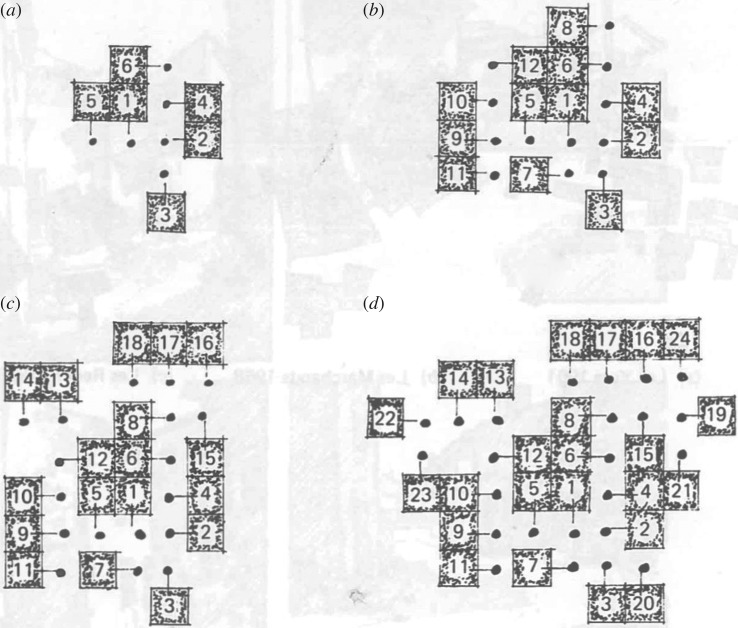
Four stages of a computer generated of a beady-ring architecture (figure 11 from Hillier & Hanson [51], p60). Reprinted with permission from Hillier & Hanson [51].

Hillier & Hanson [51] describe a rule restricted random process, involving aggregation of open space-built form dyads, that gives rise to these features. Starting with an open space-building pair, linked by the building entrance, additional dyads are aggregated by linking open space to open space (rather than building to building) (figure 6). While this generative rule gives rise to ‘phenotypic’ differences, that is differences between individual settlements, the ‘genotypic’ properties remain, such as a continuous ring of open space, fatter and thinner pieces of open space and the continuous relationship to building entrances. However, above a certain scale these settlements cease to be lifelike. In larger settlements in the region, we notice greater regularity with streets extending linearly and a deformed grid appearing.

**Figure 7.**
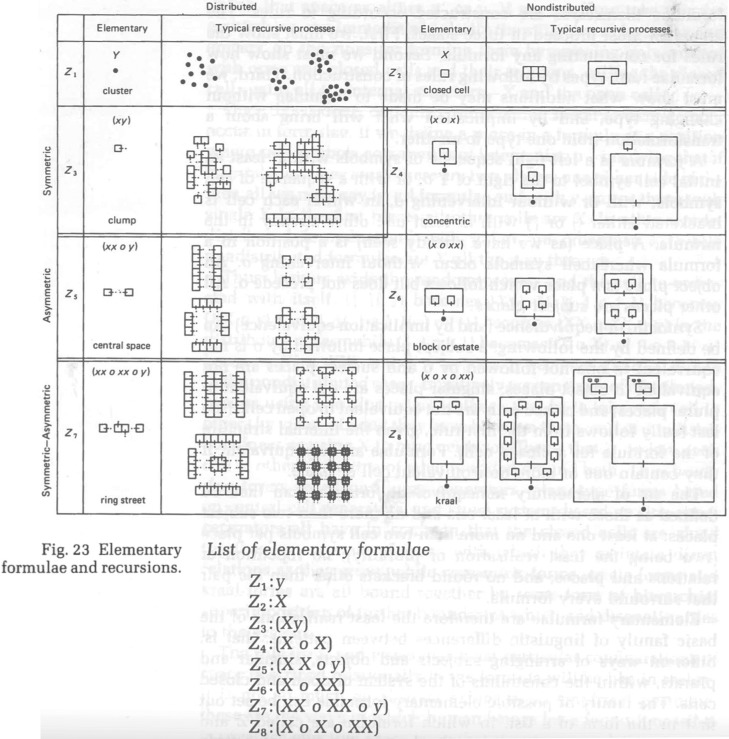
Types of recursive aggregation rules that produce a variety of settlement forms (figure 23 from Hillier & Hanson [51], p78). Reprinted with permission from Hillier & Hanson [51] (Copyright © 1989 Cambridge University Press).

Longer statements in Hillier and Hanson's recursive language give rise to more ordered spatial systems including central ‘squares’, and axially extended streets and grids. The notions of linear extension and of convexity of space are shown to emerge from rule restricted random processes (figure 7).

**Figure 8.**
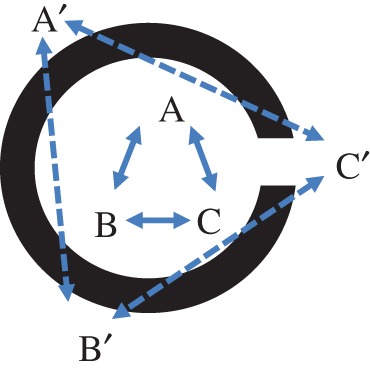
Interactions of individuals are constrained by the morphology of walls in their environment. (Online version in colour.)

These properties of linear extension and convexity carry direct social consequences. Since we are interested in what can be constructed that potentially has a systematic effect on social outcomes, we can restrict our consideration to some specific aspects of what it is possible to build. For example, we might consider that everything that one can do to configure space becomes meaningful in terms of some specific mode of perception. Thus, for the modality of human vision the constraint imposed by construction of a wall is to obstruct long distance lines of sight and movement. The way that walls are configured—that is constructed with relation to each other—affects the inter-visibility of points in space. For example, if walls are constructed to create an enclosure, the effect is to define two regions in the floor plane: those inside the enclosure and those outside. Points inside the enclosure have the significant property that if point A can see B and B can see C, then A and C can also see each other. The same does not hold for any three points in the exterior region. Here it will always be possible for A’, B’ and C’ to be located so that the walls of the enclosure hide one or another pair of points from each other. Within a sufficiently small distance of the enclosure it is possible that none of the three points can see each other. For the modality of hearing however the effect is different. Sound can travel around corners and so, in principle, it is possible for A’, B’ and C’ although not inter-visible, to be within earshot (figure 8).

The effects described above result from the relationships between stationary individuals (A, B and C), and their individual relations to the boundary of the environment.

**Figure 9.**
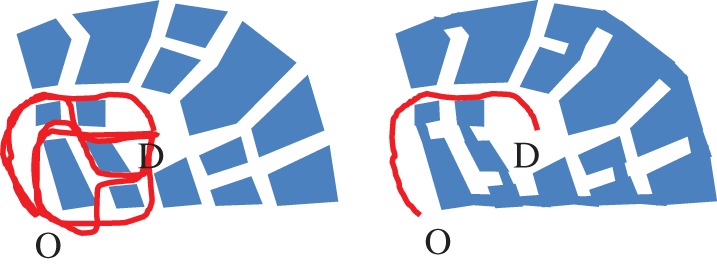
Movements of individuals in a ringy and tree-like settlement. (Online version in colour.)

There is a second kind of effect that applies when we consider mobile individuals, and how their patterns of movement bring them together, or keep them apart, in space. Consider, for example, the effect of changing the aggregation rule for the beady-ring settlement from ‘open-space links to open-space’ to ‘building links to building’. The result is a settlement characterized by tree-like spaces and cul-de-sacs. Here the configuration of the environment determines the network properties of the space, and this imposes strong constraints on patterns of movement. These in turn affect how individuals are brought into proximity as they move through the environment. For example, if the network is ‘tree-like’ there will be just one route between any origin O and destination D, however if the network is ‘ringy’, then there may be numerous different routes between O and D (figure 9).

These properties have direct effects on the probability that any two individuals will be co-present in space. They also have a direct effect on which spaces are more likely to carry movement and which will host higher numbers of co-present individuals. In this way, the spatial configuration of the environment would be expected to exert a probabilistic effect on co-presence and awareness, and, all other things being equal, to constrain and give a pattern to the probability of social interaction.

## 8. Perception and the umwelt of species

We have now come to the largest pernicious duality: are humans distinctive from the rest of living nature? This prompts the question: if so, how? The answers to these questions colour our perception of architecture, human and otherwise, and how they relate to one another. These perceptions, in turn, cast the whole premise of biomimetic architecture into a new and critical light.

The basic premise of biomimetic architecture is that humans have strayed from the basic principle that shapes the rest of living nature, namely evolution through Darwinian natural selection. This has honed living nature to a high degree of efficient use of energy and materials: ‘billions of years of research and development’, as the literature of the Biomimicry Institute vividly puts it. This prompts the question: are human constructions so radically different in form and process that mimicking the rest of living nature should even be a consideration? Or is there some fundamental unity in both human and natural built environments?

The three examples we have outlined—swarms of artificial agents, cognitive swarms of termite builders and generations of beady-ring settlements—offer different perspectives to these questions. Networks of artificial agents, for example, evince emergent properties that are the result of rules of association: algorithms. Inputs to these algorithms of construction are information from the environment, which include both built structure and other agents. Outputs are emergent phenomena of architecture and self-organization. This perspective is closely in line with biomimicry's conception of nature as selectively-honed perfection. Agent-based models consist of machines that behave according to particular suites of input/output relations. In any particular environment, these rules either work well or they do not. To the extent that there is genetic variation in these rules, natural selection will enhance the persistence of some of these variants and diminish the persistence of others. Endless repetition of this process produces the ‘billions of years of research and development’. There is no place in this scheme, however, for the agents except as vehicles for implementing algorithms: the agents do not ‘know’ whether they are constructing an apt environment, because they incapable of ‘knowledge’. They are mere machines.

In contrast, both termite swarms and beady-ring settlements evince a fundamental element of cognition, self-awareness and intentionality that is lacking from automaton agent–based models. Swarms of termite builders, for example, draw the built environment into a kind of physiological conspiracy. The mound is an extension of the termite swarm superorganism, managing flows of matter and energy between swarm and environment in the same way that, say, the intestinal epithelium does. Termites not only sense the environment and communicate and influence one another's behaviours through their own sensory and cognitive systems, they construct a mound that modulates and encodes information as would, say, sensory structures in the termites themselves. The mound is an expression of cognition as much as the human eye is an expression of the visual cognitive system. Beady-ring settlements, for their part, develop according to the inhabitants' cognitive perceptions of the built environment, which are shaped to expand perception of the environment, including lines-of-sight and acoustic channels, determined in part by visual fields in which linear extension and forward motion come together. Linear street systems and corridors are the spatial counterpart of this set of perceptual competences.

It follows that the construction of the built environment will reflect mostly the perceptual capabilities of the agents, rather than the perpetual fine tuning of the endless beta versions of the algorithms that shape the behaviours of agent swarms. The built environment thus combines Uexküll’s umwelt and innenwelt [52]. Termite swarms, for example, inhabit a much different perceptual world than humans. They have an entirely different suite of sensory capabilities—no vision, a rich chemical language, acute vibration perception, hyper-sensitivity to temporal perturbation in the environment—and this results in a very different built environment from those constructed by humans. For their part, humans have well-developed senses of binocular vision and binaural hearing that serves a sophisticated spatial perception. What is striking is not so much the differences in architecture—stark as they are—but their similarity: both are cognitive expressions of an extended organism. Termites and their constructed environments on the one hand, communities and their constructed environments on the other.

To a first order of approximation, the behaviour of a mobile individual subject with different perceptual competences will be constrained by the morphology of their environment as a function of their location, orientation, trajectory and speed of movement, their modes of perception (sight, hearing, touch, smell etc.), and the way these are integrated, the perceptual affordances of their anatomy (for example the acuity of vision, or angle of their visual field and mobility of their head), and the configuration and properties of the environment's boundaries [53].

In a social context, where numerous individuals inhabit the same environment at the same time, the location, behaviour and relationships to and between other individuals within the subject's field of awareness (each of whose behaviour is also a product of these constraints) must be added.

Finally, the individual's interaction with their environment, including that afforded by other individuals and groups, for higher animal species, must be thought of as passing through (at least) cognitive, affective and conative ‘filters’. Thus, an individual's ‘beliefs’ about the world they perceive, their ‘desires’ and emotional state, and their immediate and longer-term ‘intentions’, will all affect how they interpret and respond to their perceived environment. At a social level, the functional programme or regime of a community or organization, and community culture and power relations can be thought of as contributing to this as well.

As a first-order approximation, this is of course a reductive model. The reality will be much more complex due to the feedback loops involved, the fact that individuals have memory and learn, and that, for humans at least, organizations and communities also develop social practices over time. The effect of cognitive, affective and conative filters must also be highly dependent upon previous experience and learning, something that would be expected to vary from subject to subject according to the social and cultural context of that individual's life experience.

In humans, as technologies have been invented, these have led to an elaboration of the human umwelt. Fundamental to this have been symbol systems and their manipulation. Written language and currency have led to socially stored memory and the creation of law and economic life, along with the apparatus of politics and the state. Written history and mythology have enabled both the great religions of the world and conceptions of nationhood, and so have also been instrumental in the development of the modern state. Mathematical notation has allowed the exploration of logical inference and of abstract or hypothetical worlds, and so the development of science. Science in turn has enabled new technologies with these making possible new forms of social structure. All of these have had direct impacts on behaviour; however, behaviour in turn produces social structure. It seems to be this series of feedback loops between different strata of the social that creates the human umwelt.

The specific contribution of this paper, in questioning the ‘organism–environment’ dualism, is to consider the built environment as an active element of this model, rather than a passive background to social action, or a merely cultural artefact whose social relevance is as a carrier of meaning. Unlike technologies that act purely as symbol systems and serve primarily to communicate meaning, the built environment also acts directly upon social relations. It should be noted that humans attribute symbolic meaning to almost everything they encounter, animate, inanimate, natural or artificial. In this the built environment is no different, and so also plays an important role in the communication of meaning, both intentionally on the part of its authors and as interpreted by its users. Our point here is that the built environment is more than this in that it also acts to make possible, or to inhibit, social relations themselves.

In drawing on Turner's notion of the termite nest forming part of the physiology of the ‘super organism’, we would draw by analogy here to human society. It would seem that the built environment, rather than affecting the physiology of a human superorganism, may affect its capacity in terms of distributed cognition. It is clear that the buildings and settlements we construct are the product of a set of social processes; that they are constructed by individuals and groups all subject to the perceptions and interactions described above; and, therefore, that they record in their configuration aspects of the social forms that generated them. It is also clear that through the mechanisms of awareness afforded by inter-visibility and co-presence resulting from effects of configuration on movement routes, the built environment also holds the potential to generate and constrain social interactions and connections. In other words, it can act to reproduce a social form, or alternatively to generate new social forms.

It has not escaped our notice that this mechanism may help account for how it is that human social, cultural and technological evolution accelerated so rapidly after the first dense built settlements started to be constructed in the 10th millennium BC in the Eastern Mediterranean.

## Data accessibility

This article has no additional data.
